# Interoceptive regulation of skeletal tissue homeostasis and repair

**DOI:** 10.1038/s41413-023-00285-6

**Published:** 2023-09-05

**Authors:** Yao Xiao, Changhao Han, Yunhao Wang, Xinshu Zhang, Rong Bao, Yuange Li, Huajiang Chen, Bo Hu, Shen Liu

**Affiliations:** 1 Department of Orthopaedics, Shanghai Jiao Tong University School of Medicine Affiliated Sixth People’s Hospital, 600 Yishan Rd, Shanghai, 200233 PR China; 2 Spine Center, Department of Orthopedics, Changzheng Hospital, Naval Medical University, Shanghai, 200003 PR China

**Keywords:** Bone, Neurophysiology, Pathogenesis

## Abstract

Recent studies have determined that the nervous system can sense and respond to signals from skeletal tissue, a process known as skeletal interoception, which is crucial for maintaining bone homeostasis. The hypothalamus, located in the central nervous system (CNS), plays a key role in processing interoceptive signals and regulating bone homeostasis through the autonomic nervous system, neuropeptide release, and neuroendocrine mechanisms. These mechanisms control the differentiation of mesenchymal stem cells into osteoblasts (OBs), the activation of osteoclasts (OCs), and the functional activities of bone cells. Sensory nerves extensively innervate skeletal tissues, facilitating the transmission of interoceptive signals to the CNS. This review provides a comprehensive overview of current research on the generation and coordination of skeletal interoceptive signals by the CNS to maintain bone homeostasis and their potential role in pathological conditions. The findings expand our understanding of intersystem communication in bone biology and may have implications for developing novel therapeutic strategies for bone diseases.

## Introduction

The mammalian skeleton is a weight-bearing structure composed of intricately connected bones that provide a stable internal environment for mineral storage and functional hematopoiesis. Bone is a dynamic hard tissue with a tertiarily-mineralized extracellular matrix and robust self-renewal capacities, undergoing a process of continuous bone cell replacement via various mesenchymal progenitors, known as bone remodeling. This ongoing cellular synchronization of bone remodeling and reconstruction involves interactions among erythropoietic cells, stromal cells in the bone marrow, and bone cells. It is widely recognized that OCs and OBs play essential roles in bone remodeling, wherein new bone tissue replaces old bone through bone-forming OBs and bone-resorbing OCs.^1,2^ As OCs and OBs constantly function at their local bone remodeling sites, they require a precise central functional orchestration. Consequently, bone remodeling is closely linked to elaborate neuronal control. Any disturbance in neuronal function can impact bone homeostasis.^3^ For example, excessive chronic stress is often associated with osteoporosis, and clinical neuronal disorders such as total brain injury can result in aberrant bone metabolism.

The hypothalamus, located in the forebrain, its normal neuronal function is essential to maintain internal homeostasis which indicate that the CNS may also regulate bone dynamics. The hypothalamus coordinates internal organ information through signals from the arcuate nucleus (ARC) projected to the dorsomedial hypothalamus (DMH), paraventricular nucleus (PVN), lateral hypothalamus (LHA), and ventromedial hypothalamus (VMH).^4^ The ARC’s semi-permeable blood-brain barrier allows neurons to be exposed to circulating factors like leptin, modulating neural signals and releasing endocrine factors affecting fat, gut, bone, and peripheral tissues.^4^ The ARC also controls energy homeostasis through anorexigenic proopiomelanocortin (POMC) and cocaine- and amphetamine-related transcript (CART) neurons, as well as orexigenic neuropeptide Y (NPY) and agouti-related peptide (AgRP) neurons.^5^ The CNS plays a pivotal role in bone homeostasis through hormones and cytokines like leptin, insulin, adiponectin, irisin, osteoglycin, lipocalin 2, and osteocalcin.^6^ These hormones and cytokines respond to hypothalamic neuronal signal and subsequently manipulate energy homeostasis, satiety, and are also responsible for the central regulation of bone density and layered features. Central neuronal regulation of bone occurs through hormonal signals produced by hypothalamic neuroendocrine neurons, which are responded to by the pituitary. Additionally, increasing evidence indicates that the CNS directly modulates bone metabolism through bioactive neuronal secretions from the hypothalamus and the autonomic nervous system.^7,8^

Neurons in the peripheral nervous system, originating from sensory and autonomic ganglia, serve as relay stations for internal information transmission. These neurons transmit commands to the skeleton after the CNS collects and integrates information from the internal environment.^9,10^ The cell bodies of bone-specific primary sensory neurons reside in dorsal root ganglia (DRG) along the spinal cord and cranial nerve ganglia, extending their axons to target bone tissues.^10^ The regulatory potential of sensory neurons on bone is well-documented through the release of calcitonin gene-related peptide (CGRP), substance P (SP), nerve growth factor (NGF), and semaphorin 3 A (Sema3A), which influence bone cells via vasodilation, trophic function, and direct effects.^11–13^ Autonomic nerves, on the other hand, respond to stressors to maintain internal homeostasis, such as energy consumption and circadian clock rhythms. These signals are responsible for sympathetic and parasympathetic outflow and, consequently, the regulation of bone metabolism.^10,14,15^

The above mentioned internal information, termed interoception, can be described as the perception and regulation of the body’s internal physiological and metabolic states, such as energy homeostasis, blood pressure, gut motility and bone density through communication and integration between the CNS and peripheral organs.^16,17^ The CNS perceives and regulates the skeletal system via nonconscious sensing, with bone interoception acting as an information bridge that connects the CNS and peripheral skeletal organs. The skeletal system continuously undergoes remodeling and metabolism to adapt to various circumstances, such as weight-bearing, aging, or disorders.^18–20^ Skeletal interoception mediates this process nonconsciously.

Through interoceptive modulation, the CNS gathers information from both external and internal environments, enabling communication and connections between all organs and tissues within the body through efferent autonomic and afferent somatic pathways.^21^ Since bone is one of the largest organs in the human body, many factors contribute to maintaining its homeostasis. Substantial clinical observations have demonstrated a bidirectional crosstalk between the brain, nervous system, and bone. For example, neurodegenerative disorders are associated with osteoporosis, epilepsy, schizophrenia, post-traumatic stress disorder, depression, stroke, Alzheimer’s disease, and Parkinson’s disease.^22–28^ Moreover, rare genetic skeletal diseases manifest with impaired brain development, like haploinsufficiency of *Runx2*, which is responsible for cleidocranial dysplasia, and often accompanied by delayed brain development and late-onset progressive cognitive decline.^29,30^ Trauma events particularly highlight the importance of the crosstalk between the nervous system and bone. CNS trauma, including brain and spinal cord injuries, promotes bone regeneration,^31,32^ whereas peripheral bone injury negatively affects or aggravates brain injury.^33^ To illustrate, CNS injuries have been determined to cause heterotopic ossification in more than one in five patients, implying a regulatory role for the nervous system in bone homeostasis^34^ and supporting the CNS’s involvement in maintaining skeletal homeostasis.^35^ Existing reviews have comprehensively described the neuronal regulation of bone homeostasis and repair, including hypothalamic control of bone metabolism,^3^ impact of the autonomic system on the skeleton,^9^ crosstalk between energy metabolism and bone remodeling,^6^ and neuromodulation of bone.^1,36,37^ However, interoceptive regulation of skeletal tissue homeostasis and repair is an emerging area of skeletal research lacking a thorough understanding and review.

Although a substantial body of evidence underlies the neuromodulation of bone, how the nervous system senses intraosseous homeostasis within skeletal interoception, provides feedback, and accurately controls the neuromodulation of bone is poorly understood. Skeletal interoception can be described as skeletal sensory nerves perceiving the state of intraosseous homeostasis, transmitting signals from the bone through sensory nerves, dorsal root ganglia, and superficial dorsal horns of the spinal cord to the CNS (specifically the hypothalamus). The CNS then integrates and interprets these signals as an executor, ultimately relaying a bone response via descending autonomic nerves and neuroendocrine regulation to maintain homeostasis.^38–41^ Therefore, this review focuses on how sensory afferents mediate interoceptive signals to facilitate central regulation of skeletal homeostasis, central processing of interoception to coordinate autonomic nerve regulation of skeletal homeostasis, and how the hypothalamus processes interoceptive signals to maintain skeletal homeostasis. Ultimately, this review aims to support a comprehensive understanding of how the CNS generates and coordinates skeletal interoceptive signals and how skeletal interoception regulates bone homeostasis through intricate intracellular and extracellular signaling pathways, particularly the regulatory mechanisms mediated by the hypothalamus. Furthermore, we also discuss the role of skeletal interoception in bone fracture healing, and its association with specific pathological conditions, such as the potential mechanisms behind traumatic brain injury (TBI)-promoted fracture healing.

## Sensory afferents mediate interoceptive signals to facilitate central regulation of skeletal homeostasis

The specific mechanisms through which interoceptive sensory signaling from peripheral organs triggers a central-to-autonomic nerve response are largely unclear. In this section, we focus on CNS involvement in the maintenance of skeletal homeostasis through skeletal interoception. In doing so, we discuss sensory innervation of human bone, the localization of neuroreceptors in the skeletal microenvironment, how prostaglandin E2 (PGE2) and cyclooxygenases 2 (COX2) influence skeletal tissue metabolism and inflammation, and the critical mediating role of PGE2/EP4 signaling. We close this section by describing how sensory nerves mediate PGE2 signals involved in fracture and healing.

### Sensory innervation of the skeleton

Sensory nerves, extending from the DRG of the spinal cord, innervate various tissues throughout the body, including skin, bone, joints, tendons, and muscles.^9^ These nerves generate proprioception signals, detect noxious stimuli, interpret pain, and recognize changes in external factors, such as thermal and tactile stimuli and inflammatory cytokines. These relay signals are then processed and integrated in higher brain centers.^42–44^ In bone, sensory fibers primarily innervate blood vessels in the bone marrow and periosteum, with those located in cortical and trabecular bone structures sensitive to mechanical stimuli. Few sensory nerves are found in mineralized bone.^1,45,46^ Sensory fibers detect pain sensations typically associated with trauma, fractures, bone malignancies, and potentially genetic diseases like osteogenesis imperfecta. These fibers are elongated and linear, encompassing both heavily and lightly myelinated A-fibers and peptide-rich C-fibers.^9,46,47^ Sensory nerves are distributed at varying densities in the periosteum, bone marrow, and cortical bone with an estimated ratio of 100:2:0.1.^48^ However, the bone marrow contains more sensory nerves than the periosteum due to its larger area.^49^ Furthermore, sensory nerve fibers are more densely innervated in the epiphysis compared to the metaphysis, and are mainly restricted to vascularized CD31^+^ Haversian canals.^1,50^

**Fig. 1:**
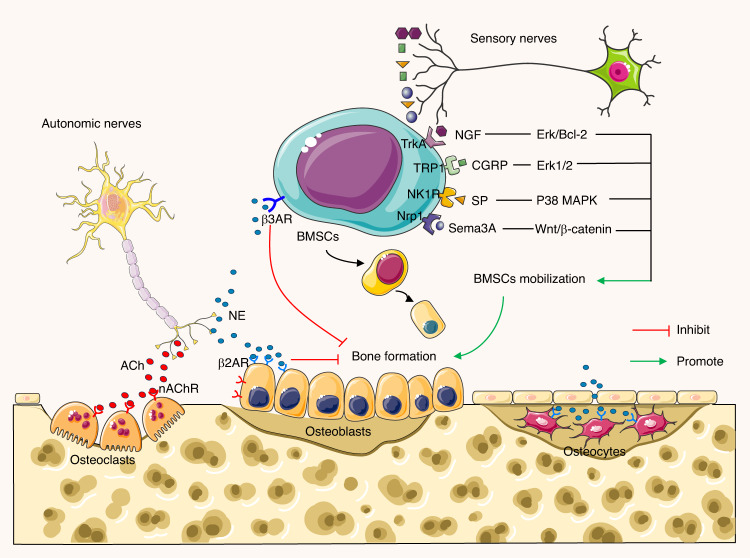
Sensory afferents coordinate autonomic efferents to regulate bone metabolism. The autonomic nerves consist of two antagonistic arms: parasympathetic cholinergic nerves and sympathetic adrenergic nerves. Cholinergic nerve fibers secrete acetylcholine (ACh), which mainly acts on osteoblasts to favor bone mass accrual. Parasympathetic neurons may also suppress sympathetic signaling in an M3R-dependent manner. Sympathetic adrenergic nerves release norepinephrine (NE), which affects osteoblasts and osteocytes via β2AR to inhibit bone formation. NE also inhibits bone marrow mesenchymal stem cells (BMSCs) migration to bone formation and osteogenesis stimulated by β3AR through the JNK pathway. Sensory nerves promote BMSCs mobilization and bone formation through NGF, CGRP, SP, and Sema3A via corresponding receptors in different pathways

Sensory nerves can be classified by their receptors and channels, such as toll-like receptors, tyrosine kinase receptors, transient receptor potential ion channels, and mechanosensitive Piezo channels.^44,51^ These receptors and channels initiate intracellular signaling and the release of neuropeptides.^44^ In bone, a substantial proportion of thinly myelinated and unmyelinated sensory nerves express neurotrophic receptor tyrosine kinase type 1 (TrkA), which has a high affinity for NGF.^46,52^ NGF is closely associated with primary and secondary ossification in endochondral bone formation.^44,53^ Additionally, innervation density increases near bone remodeling sites and within cortical pores in humans.^54^ The sensory innervation of nerve fibers and localization of neurotransmitter receptors in the skeletal microenvironment play a crucial role in bone metabolism. Sensory nerves regulate bone metabolism through multiple neuropeptides, as described in Fig. 1.

### Bone utilizes PGE2 as an interoceptive signal to the CNS

Sensory afferents mediate interoceptive signals usually initiated by endogenous molecules secreted in bone tissue and recognized by their receptors on the sensory nerve. Studies have found that prostaglandins and cyclooxygenases can transmit biological changes from bone tissues to sensory nerve fibers, which are known to influence skeletal tissue metabolism and inflammation.^55,56^ PGE2, one of the most versatile prostaglandins, is regulated by the limiting enzyme cyclooxygenase.^57^ PGE2 has been shown to affect nociceptor sensitization and primary pain generation. Non-steroidal anti-inflammatory drugs and selective COX2 inhibitors are two common medications for treating musculoskeletal pain.^58^ In terms of bone metabolism, several multicenter studies have found that COX2 selective inhibitors can decrease men’s bone mineral density (BMD) and protect postmenopausal women from BMD loss related to menopause.^59,60^ This suggests the involvement of PGE2 in bone mineralization. Another study found that local administration of PGE2 promoted bone formation, and PGE2 levels increased following bone fractures;^56^ inhibiting PGE2 strongly delayed fracture healing.^61,62^ Lastly, hydroxyprostaglandin dehydrogenase 15 (HPGD) mutant mice exhibit higher levels of PGE2, which has been shown to effectively stimulate regeneration in various tissues in vivo.^63^ Interestingly, patients with HPGD mutations have been found to develop new bone beneath the subperiosteum.^64^

Osteoblast receptor EP4 is thought to be responsible for the anabolic effect of PGE2 on bone formation. Studies have shown that knocking out prostaglandin E receptor 4 in osteoblastic cells does not impair bone density, suggesting that PGE2 does not directly promote bone formation through osteoblasts.^55^ Pathological conditions of bone loss, such as those related to aging or menopause, are often accompanied by impaired sensory nerve function and elevated PGE2 levels. PGE2-induced pain may potentially result from sensory nerve activation, transmitting signals promoting bone density to the brain. In this context, Chen and colleagues demonstrated that sensory nerves could perceive bone density through a local sensor, PGE2, delivered by osteoblasts to sense internal signals for maintaining bone homeostasis. These findings indicate that the interoception of the skeletal system closely links the local environment with the CNS, primarily controlled by PGE2/EP4 signaling via sensory nerves.

### Sensory nerves transmit skeletal interoception signaling to the CNS

DRG sensory nerves innervate tissues throughout the body, relaying information related to proprioception and nociception.^65^ After receiving PGE2/EP4 signals from sensory nerve endings, afferent nerves relay the signals to the hypothalamus, initiating physiological responses within the CNS. In this way, sensory nerves serve as a transmitter of skeletal interoception signaling to the CNS. Cholinergic signaling in bone is widely recognized for its role in bone remodeling, as is central acetylcholine (ACh) signaling, which is supported by robust genetic and pharmacological evidence.^66^ In the spinal afferent system, visceral interoception is transmitted to the CNS via afferent pathways.^67^ Research also indicates that the skeletal system interacts with the CNS to maintain homeostasis through sensory afferent and sympathetic efferent pathways.^68,69^ Ultimately, it seems that sympathetic nerves tightly control bone remodeling microenvironments through precise temporal-spatial actions, as well as energy and calcium metabolism associated with bone formation.^70,71^ These processes are likely coordinated by the CNS, and such precise modulation of bone remodeling may be attributed to simultaneous interoceptive inputs from bone remodeling microenvironments.

### Sensory nerve transmission of interoceptive signals mediates central regulation of bone fracture healing

Patients with sensory dysfunction often experience bone loss and a high fracture rate. For example, congenital insensitivity to pain and anhidrosis have been associated with tibia fracture in pseudoarthrosis.^72^ Furthermore, nerve injuries and neuropathy are strongly correlated with disrupted serum PGE2 levels.^73^ Local PGE2 levels significantly increase after bone fractures, and PGE2 inhibition impairs bone healing.^62^ These findings suggest that sensory nerves mediate PGE2 signals involved in fracture and healing. A previous study confirmed that osteoblast-derived PGE2-EP4 interoceptive signals act on advilin-positive ascending sensory nerves, transmitting mechanical signals to the hypothalamus, which then regulate the commitment of Lepr^+^ bone mesenchymal stem cells (MSCs) by toning down descending sympathetic activity.^56^ Furthermore, mechanical load or PGE2 degradation enzyme inhibitors activate skeletal interoceptive signals, accelerating osteogenesis of Lepr^+^ stromal cells and promoting bone healing after fractures.^56,74^ Additionally, EP4 receptor activation stimulates bone formation and prevents bone loss.^75^ Deletion of EP4 in sensory nerves decreases osteogenesis and inhibits the differentiation ability of MSCs.^56^Therefore, osteoblast-derived PGE2 promotes bone formation by activating EP4 in sensory nerve regulation of MSCs, although other sensory nerve secretory neuropeptides, such as substance P and CGRP, may also be involved in regulating MSCs activity.

## Central processing of interoception to coordinate autonomic nerve regulation of skeletal homeostasis

After receiving afferent interoceptive signals from the sensory nerves, the CNS interprets and generates the regulatory signals. Efferent interoceptive signals are then relayed back to bone tissue via neuroendocrinal regulation and the autonomic nervous system to modulate skeletal homeostasis. In this section, we examine autonomic innervation of the skeleton and discuss how autonomic nerves respond to central interoception to regulate bone metabolism via the norepinephrine (NE)/androgenic receptor (β-ARs) pathway and the role of downstream interoceptive autonomic nerve regulation in promoting bone fracture healing.

### Autonomic nerve innervation in the skeleton

The autonomic nervous system comprises the sympathetic nervous system (SNS) and the parasympathetic nervous system (PNS). Autonomic nerve fibers are widely distributed throughout the body and are well-known for modulating involuntary functions that maintain homeostasis, including respiration, circulation, digestion, and stress.^76–78^ Sympathetic and parasympathetic neurons are found within the skeletal system, including subchondral bone, bone marrow, periosteum, and synovium.^76,79–81^ These autonomic nerve fibers are small, unmyelinated neurons, similar in size to C fibers.^80^ Both the SNS and PNS play crucial roles in bone remodeling, with outflow stimulated by energy expenditure, circadian rhythms, and stress.^14,15,82^ Alterations in autonomic outflow or interventions may affect skeletal homeostasis.^9^

Postganglionic autonomic neurons can be characterized by various immunoreactive proteins, such as small molecule neurotransmitters and enzymes.^83^ The SNS is characterized by the expression of tyrosine hydroxylase, β-hydroxylase, and norepinephrine transporter (NET).^9,84,85^ SNS synaptic terminals can co-release NE and NPY.^83,86^ Bone cells, covered with α- and β-adrenergic receptors (α-AR and β-AR), can identify and respond to NE. NE is reabsorbed and catalyzed by the enzyme monoamine oxidase (MAO), which has been found within osteocytes, responding to SNS activity.^86,87^ The PNS, on the other hand, can be characterized by immunoreactivity to ACh, vesicular ACh transporter, choline acetyltransferase, acetylcholinesterase, and vasoactive intestinal peptide (VIP).^9,88,89^ In response to PNS activity, activated nicotinic and muscarinic ACh receptors (nAChR and mAChR), expressed on the surface of bone cells, regulate cholinergic responses to modulate the skeletal system.^90,91^ Cholinergic signaling pathways significantly influence osteogenesis through increased osteoblast proliferation, stem cell differentiation, and physiological processes in bone cells, leading to upregulated bone formation; these effects are summarized in Fig. 1.

### Autonomic nerves respond to central interoception to regulate bone metabolism via norepinephrine release

Autonomic nerves release neuropeptides such as NPY, VIP, ACh, and NE from synapses that diffuse through bone, joint, and muscle to reach cells within the skeletal system and exert their effects. The autonomic nervous system should be able to stimulate bone cells via direct contact, but few studies have explored this due to difficulty of observation. Consequently, research on the regulatory effects of the autonomic nervous system on bone homeostasis tends to focus on neurotransmitter outflow and the administration of sympathetic agonists and antagonists, which can reflect the activated autonomic role.

NE is the canonical neurotransmitter of postganglionic noradrenergic sympathetic nerves.^10^ Bones consistently subjected to mechanical stress exhibit higher metabolic rates associated with angiogenesis and sympathetic nerve innervation.^92–94^ Sympathetic nerves modulate the metabolism and function of OBs and OCs through α- and β-adrenergic receptors (ARs) influenced by sympathetic outflow and adrenergic receptor signaling^95–97^ as summarized in Fig. 1. βAR subtypes are the primary receptors contributing to bone remodeling due to their abundant expression on bone cells compared to α-AR subtypes.^98^ Specifically, β2AR subtypes are more widely expressed on osteoblastic cell lines, with low expression of α1AR and α2AR.^96,99,100^ β2ARs are also expressed on OCs, but few studies have investigated their function upon stimulation.^87^ Both α and β isoforms of catabolic enzymes MAO are detectable in both osteoblast precursor cells and differentiated OBs, as well as NET in differentiated OBs, allowing the bone cell to intake and catabolize NE.^86,87^

β-AR blockers, such as propranolol, can increase vertebral trabecular bone mass and prevent estrogen-induced bone loss.^15,101,102^ Conversely, studies have also shown that the β1AR agonist dobutamine enhances cancellous bone mass, suggesting that β1ARs have a conflicting role in maintaining OBs function as compared to β2ARs.^103,104^ In β2AR-deficient mice, trabecular bone mass increased, accompanied by reduced bone resorption and enhanced bone formation.^15,105^ This phenomenon was similar to the results observed with β2AR inhibition using β-AR blockers. However, in contrast to the findings of human studies, β1AR knockout led to low bone mass in mice femurs and a reduced anabolic response to compression loading. A murine study that globally knocked out all three β-ARs showed elevated trabecular bone mass,^106^ suggesting that β-ARs predominantly reduce bone mass. However, the specific phenotype primarily regulating this process remains understudied. One murine study found that the deletion of both β1ARs and β2ARs resulted in a low bone mass phenotype,^107^ possibly indicating that β1ARs dominate β2ARs. A clinical trial by Khosla et al. observed increased bone formation after administering the β1AR selective blocker nebivolol to postmenopausal women.^108^ β1-AR selective blockers facilitated bone mass, reducing bone resorption levels and increasing bone density of ultra distal radius tissue. This result provides evidence for the potential clinical application of β1AR blockers in regulating bone reconstruction. However, the underlying mechanism for the conflicting outcomes of human and rodent bone metabolism requires further investigation. As for α-ARs, global deletion of α2A/α2CAR in female mice led to high bone mass, accompanied by lower bone resorption and higher bone formation.^98^

The assessment of sympathetic outflow activity is crucial when considering the role of sympathetic receptors in bone metabolism. Mice lacking Dbh, a vital component of NE production, exhibited high trabecular bone mass.^15^ Another study found that mice lacking FoxO1 in Dbh-neurons presented with low sympathetic outflow and high bone mass.^84^ Chronic immobilization stress, a method to promote endogenous sympathetic outflow, did not induce bone mass loss by itself but did so when combined with reboxetine, an NE reuptake blockade.^86^ This might suggest that chronic SNS stimulation has limited independent effects on osteocyte metabolism.

The mechanisms by which isoproterenol (a surrogate of sympathetic signaling) or NE affect bone remodeling primarily involve increasing the pro-osteoclastogenic cytokine RANKL in OBs, triggering osteoclastogenesis and ultimately inducing bone resorption.^105^ Sympathetic stimulation not only enhances osteoclastogenic activities but also modulates bone formation by inhibiting OBs proliferation.^100,109,110^ Further, a study found that the expression of β2ARs in propranolol-cultured OBs was significantly reduced, accompanied by increased BMP2, Runx2, COL-1, and osteocalcin, promoting osteogenic differentiation of OBs and MSCs.^111^ Moreover, in vivo examination demonstrated that propranolol enhanced implant osseointegration.^108^ Overall, β2ARs are the primary mediators of Ach regulation in the skeletal system, inhibiting bone formation and increasing RANKL expression, and leading to osteoclast formation and increased bone resorption. These findings clarify the predominant effect of blocking β-ARs in promoting bone formation, indicating potential applications in clinical therapy, as described in the previous section.

The SNS is also affected by glucocorticoid metabolic signaling. In a conditional knockout of the glucocorticoid receptor (GR-CKO) mouse model, bone loss was evident, accompanied by increased fat in bone marrow. However, the application of propranolol, a competitive antagonist to β-adrenergic receptors, significantly increased cortical bone mass in GR-CKO mice during caloric restriction.^112^ Although propranolol did not reverse CR-induced marrow fat, corticosterone levels were elevated, suggesting a possible bone-hypothalamus-pituitary-adrenal crosstalk during metabolic stress.^113^ This finding provides new insight into the regulation of the SNS in metabolic-induced bone homeostasis. In one of our recent studies, sensory nerve regulation of mesenchymal stromal cells promoted bone formation via the inhibition of sympathetic activity, primarily modulated through the PGE2/EP4 sensory nerve axis.^56^ This result indicated a cooperative effect between the sensory and sympathetic nervous systems on bone homeostasis. Mechanical loading induces the innervation of sensory and sympathetic neurons inhabiting the skeletal system. Wang et al. recently found that mechanical loading significantly reversed β2-AR agonist terbutaline-induced bone loss,^114^ suggesting that sympathetic modulation of bone metabolism may be closely associated with the endocrine system or sensory nerve interoception relay. Given these promising findings, further investigation of this regulatory pathway is warranted.

### Processing of interoceptive signals by the CNS utilizes autonomic nerves to coordinate bone fracture healing

Bone fracture healing reflects the process of bone construction in the skeletal system. Sympathetic inhibition with propranolol administration increases mineral apposition rates and callus formation in femoral-fractured rats.^115^ While the local metabolic effects of the ANS on bone cells are known, few studies have investigated the regulatory potential of interoceptive sympathetic function on fracture healing.^9^ Activation of Adrb2 in OBs by NE administration inhibits cAMP-response element binding protein (CREB) phosphorylation, reducing OBs proliferation.^110^ Chen et al. found that PGE2-induced CREB phosphorylation in the VMH through EP4 signaling increased urine NE concentrations in EP4_Avil_^-/-^ mice.^9^ This suggests that sensory nerve activation via PGE2 signaling suppresses sympathetic tone, increasing osteoblastic bone formation and promoting bone healing.^55^ Conversely, another study found that sympathectomy using 6-Hydroxydopamine in adult mice led to lower bone volume and weaker mechanical strength during fracture healing, conflicting with the consensus on β2AR modulation’s skeletal system impact.^116^ Recently, Stephen et al. discovered that interleukin-6 (IL-6) induction causes a cholinergic switch in bone-innervating sympathetic nerves, with GDNF-family receptor-α2 and its ligand neuritin regulation mediating osteoprogenitors to promote bone formation.^117^ Inflammation upon bone fracture increased IL-6 and PGE2 at wounded sites.^118^ Taken together, these findings suggest that downstream interoceptive SNS regulation promotes bone fracture healing by inhibiting sympathetic tone and promoting parasympathetic function.

## The hypothalamus processes interoceptive signals to maintain skeletal homeostasis

**Fig. 2:**
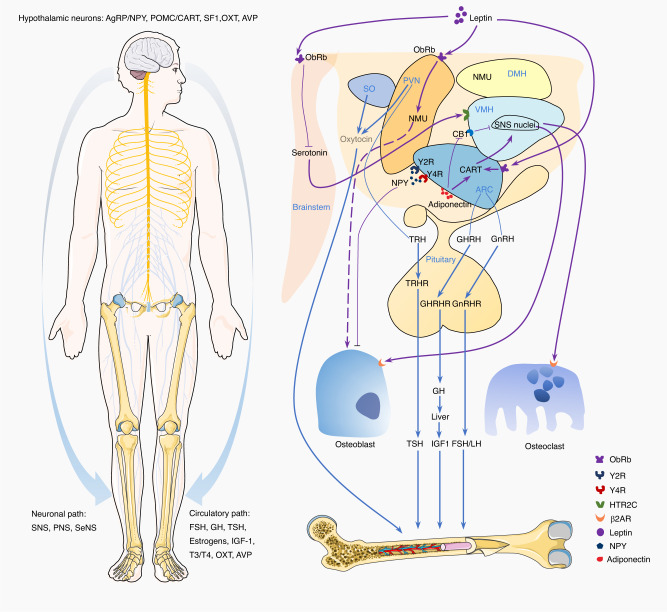
Hypothalamus-mediated interoceptive regulation of bone homeostasis through neuronal and circulatory pathways. Leptin binds to its receptors (ObRb) in the brainstem, stimulating serotonergic neurons to release serotonin, which subsequently binds to HTR2C receptors on ventromedial hypothalamus (VMH) neurons, altering bone mass. Moreover, leptin up-regulates the expression of cocaine- and amphetamine-regulated transcript (CART) in the arcuate nucleus (ARC) of the hypothalamus, inhibiting bone resorption. Additionally, neuromedin U (NMU) serves as a downstream effector of leptin-mediated modulation of bone mass at the paraventricular nucleus. Neuropeptide Y (NPY) acts through its receptors (Y2R and Y4R) in ARC to inhibit osteoblast proliferation and function. Adiponectin up-regulates CART in ARC and inhibits CB1 in VMH via hypothalamic AdipoR1 and R2 receptors. The sympathetic nervous system (SNS) regulates osteoblasts and osteoclasts via β2AR. Furthermore, the hypothalamus regulates bone homeostasis through an endocrine route involving hormones produced by the anterior pituitary gland as well as neuron-derived oxytocin (OXT) and arginine vasopressin (AVP). Involved hormones include thyroid-stimulating hormone (TSH), luteinizing hormone (LH), follicle-stimulating hormone (FSH), insulin-like growth factor-1 (IGF-1), and others. OXT is produced by supraoptic oxytocin (SO) neurons and paraventricular nucleus neurons (PVN) and is involved in the regulation of bone

The hypothalamus is a key regulator of organ, neural, and endocrine activities. It not only plays an important role in regulating body temperature, appetite, gland hormone secretion, energy expenditure, and emotional activities^4,119,120^ but also in the critical process of bone remodeling.^4,6,7^ The pathways through which the hypothalamus mediates the regulation of bone homeostasis are shown in Fig. 2. As sensory afferents mediate interoceptive signals arriving at the CNS, the hypothalamus interprets this information and sends signals to peripheral bone tissue. Hypothalamus-derived efferent interoceptive signals can reach sympathetic preganglionic neurons in the spinal intermediolateral nucleus from the first thoracic to the second lumbar spinal cord segments, and these signals can also be transferred through brainstem neurons.^9,16^ How the hypothalamus interprets and processes the afferent interoceptive signals to regulate bone homeostasis is not well understood. This section examines how the hypothalamus interprets skeletal interoception to facilitate skeletal homeostasis, including how hypothalamic VMH CREB signaling regulates sympathetic tone and how sensory to sympathetic relay via the hypothalamic VMH circuit regulates MSCs lineage commitment. It also explores the pathways that hypothalamic interoception uses to regulate the neuroendocrine system to maintain skeletal homeostasis, including relational regulation of leptin, NPY, agouti-related peptide, cocaine and amphetamine-regulated transcript (CART), and the endocannabinoid (EC) system.

### Hypothalamic processing of skeletal interoception to orchestrate skeletal homeostasis

#### Hypothalamic VMH CREB signaling regulates sympathetic tone after receiving skeletal interoceptive signals

CREB regulates gene expression programs to maintain neural development, survival, and complex adaptive responses.^121^ Through interoceptive signaling from sensory nerves, hypothalamic neurons respond via serotonin and CREB signaling to modulate sympathetic tones for bone metabolism regulation. CREB signaling has an important impact on bone homeostasis. PGE2 activates EP4 in sensory nerves and transmits skeletal interoceptive signals to the VMH. Furthermore, as sensory nerves are required for precise central regulation of skeletal homeostasis, EP4 receptors are responsible for PGE2-induced CREB signaling in the VMH. Mice treated with EP1/3 agonists showed no impact on CREB phosphorylation in the VMH, whereas mice treated with EP4 agonists exhibited significantly elevated pCREB levels, suggesting EP4 in sensory nerves is essential for CREB phosphorylation in the VMH induced by bone-derived PGE2. Moreover, the CNS controls bone homeostasis by maintaining proper local PGE2 levels during normal skeletal interoception. Xue and colleagues demonstrated that low-dose celecoxib treatment maintained PGE2 in proper concentrations and increased pCREB levels in the VMH, promoting bone formation.^122^ These results strongly support the critical role of the hypothalamus in regulating bone interoception through the CNS.

#### Sensory to sympathetic relay via the hypothalamic VMH circuit regulates MSCs lineage commitment

There is consensus that the differentiation of bone marrow skeletal stem/stromal cells (SSCs) into osteoblasts, adipocytes, and chondrocytes maintains the integrity of bones.^123–125^ Preserving SSCs balance and allowing them to commit to different cell lineages is essential for bone health.^126^ SSCs can potentially commit as progenitors, such as osteoblast progenitors and adipocyte progenitors, to further differentiate into mature osteoblasts and adipocytes. Hu et al. discovered that sensory nerves regulate MSCs fate by binding the bone-forming signal PGE2 to EP4 receptors on sensory nerves. This interoceptive signal is transmitted to the hypothalamic VMH and then relayed back via sympathetic nerves to modulate SSCs lineage commitment.^56^ Mammalian stem cells are located adjacent to sensory nerves to form niches. In sensory denervation mouse models, the number of SSCs decreased significantly, supporting the notion that SSCs fate may be under the precise control of sensory nerves. By triggering skeletal interoception through mechanical loading or by inhibiting the PGE2 degradation enzyme, the osteogenic potential of Lepr^+^ stromal cells were activated, greatly accelerating the fracture healing process.^56,74^ This research demonstrates and clarifies the mechanism by which sensory interoception, the CNS, and sympathetic relay modulate the differentiation fate of MSCs to maintain skeletal system homeostasis and regulate fracture healing.

### Hypothalamic interoception regulates the neuroendocrine system to maintain skeletal homeostasis

#### Interoception participates in Leptin’s regulation of bone metabolism

Leptin, primarily synthesized by white adipose tissue, is essential for maintaining whole-body energy homeostasis and has significant regulatory effects on bone turnover.^4,127^ Leptin is recognized by its corresponding receptor on the arcuate nucleus in the hypothalamus, effectively acting on energy balance by suppressing appetite.^128^ Initial loss-of-function studies investigating leptin signaling reveal the relationship between leptin and bone homeostasis. Leptin (*ob/ob*) or leptin receptor (*db/db*) knockout mice exhibited decreased bone mass due to reduced cortical bone formation.^4,129–131^ However, other evidence showed that *ob/ob* mice displayed a marked increase in cancellous bone volume and turnover without affecting cortical bone volume.^35^ Additionally, leptin-deficient mice exhibited shorter femur length, reduced femoral bone mineral density (BMD), thinner cortical bone thickness, and less mineralization, but increased vertebral length, lumbar BMD, and cancellous bone volume compared to the wild-type group.^132^ These studies indicate a complex regulatory network for leptin, which appears to have opposing effects on cancellous bone formation and cortical bone formation.

Leptin regulates bone homeostasis through the hypothalamus, despite the presence of leptin receptors in local OBs (Fig. 2). The intracerebroventricular (ICV) administration of leptin led to bone loss in leptin-deficiency mice suggesting leptin inhibits bone formation via central neuronal control.^35^ Leptin receptor (ObRb), highly expressed in VMH neurons, is a neuronal mediator in bone formation, with VMH neuron destruction increasing bone mass.^15^ The SNS participates in bone regeneration in a leptin-dependent manner. Local leptin injection into the VMH area activates sympathetic outflow, increasing plasma noradrenalin and adrenalin levels.^133^ Blocking β2AR prevents cancellous bone loss in leptin-deficient mice following ICV leptin infusion,^15^ while global β2AR ablation increases cancellous bone volume.^105^ These results demonstrate a CNS-managed sympathetic regulatory pathway on bone homeostasis. Recent research suggests VMH primary cilia play a pivotal role in regulating leptin resistance, maintaining OBs-OCs interplay through sympathetic tones.^134^ Leptin also affects bone formation via brainstem and serotonergic signaling. Serotonin binds to serotonin 2c receptors in the VMH and serotonin 1b receptors on OBs membranes to suppress bone generation.^135,136^ Leptin may inhibit serotonergic receptors and decrease serotonin synthesis.^135,137^

Leptin receptors have also been identified in adult primary chondrocytes and OBs, suggesting that leptin may directly influence bone growth and metabolism.^8,137^ In *db/db* mice, bone tissue displacement was associated with increased bone mass, osteoblast number, and activity without altering energy homeostasis, suggesting that leptin regulates bone metabolism through local regulatory mechanisms rather than central control.^138^ In *ob/ob* mice, long-term hypothalamic leptin gene delivery enhanced bone turnover and significantly decreased the cartilage matrix within the bone, indicating that osteopetrosis in *ob/ob* mice can be reversed by site-specific leptin supplementation.^139^ Leptin may also stimulate fibroblast growth factor 23 expression in bone to regulate growth.^140^ Other studies have shown that leptin accelerated bone fracture healing in mice by upregulating the expression of vascular endothelial growth factor (VEGF) in callus tissue.^141^ Reduction of leptin resulted in rat bone metabolism disorder during high-fat-diet-induced catch-up growth, via balancing the OPG/RANKL signaling pathway.^142^ Furthermore, leptin enhances BMP9-induced osteogenic differentiation of MSCs by activating JAK/STAT signaling.^143^ Additionally, leptin reduces cementoblast mineralization and survival via ERK1/2 activation in vitro.^144^

In summary, leptin levels are closely correlated with bone formation and BMD. Leptin can act centrally to modulate axial trabecular bone through the sympathetic nervous system, whereas peripheral leptin promotes bone remodeling and mineralization, particularly in cortical and appendicular bone.

#### Neuropeptide Y responds to hypothalamic interoception to regulate bone metabolism

NPY, a highly conserved 36-amino acid peptide, is extensively expressed in the CNS and peripheral nervous system, with abundant expression in the hypothalamic ARC.^145^ NPY stimulates endocrine activities, immune responses, and energy homeostasis^146,147^ and connects nerves and the skeletal system through central and peripheral pathways.^148,149^ In peripheral environments, NPY co-stores and co-releases with noradrenaline in postganglionic sympathetic nerves.^146^ Hypothalamic-derived NPY negatively regulates osteoblast activity and bone formation. High NPY levels increase food intake, leading to weight gain and bone mass loss.^150^ Mice overexpressing hypothalamus-specific NPY show an anti-osteogenic phenotype,^151^ while disruption of central NPY signaling increases bone mass accrual.^152,153^ Excessive NPY expression in mature OBs and OCs decreases trabecular and cortical bone volume without altering bone formation rate and OCs activity.^154^ A recent study found that autonomic nervous system regulation of osteocyte NPY controls bone marrow fat balance.^155^ Sympathetic and parasympathetic nerve terminals release norepinephrine and acetylcholine, stimulating or inhibiting NPY production in osteocytes through *β*2AR and M3 receptors, respectively.^155^ NPY binding to Y1R on osteocytes suppresses cAMP/PKA/CREB signaling, inhibiting pro-osteogenic/anti-adipogenic transcription factors *Tead1* and *Junb*, and promoting MSC adipogenesis.^155^ In summary, NPY negatively regulates bone formation and is closely associated with the CNS, but its modulatory pathways warrant further investigation.

**Fig. 3:**
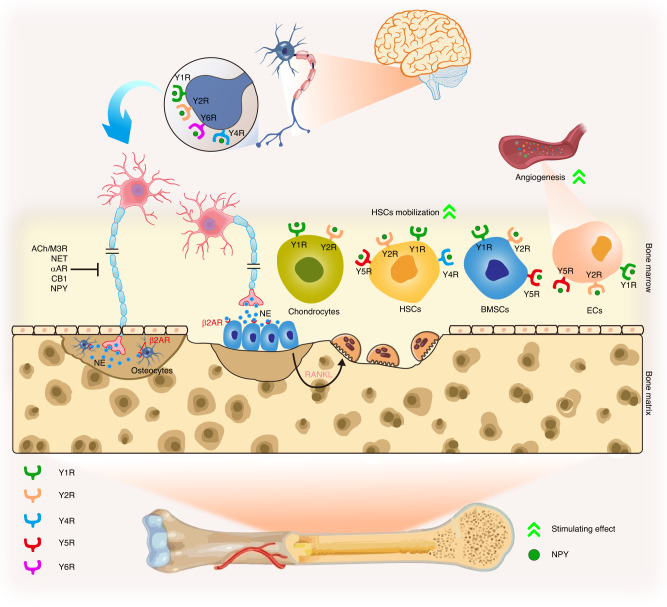
Neuropeptide Y as an interoceptive effector modulating the bone microenvironment via central and peripheral pathways with different receptors. Noradrenergic nerve terminals in bone secrete norepinephrine (NE) near osteoblasts and osteocytes, stimulating the β2AR, subsequently inhibiting bone formation and up-regulating RANKL expression, leading to osteoclast formation and increased bone resorption. This signal is controlled by neuropeptide Y (NPY), the endocannabinoid system, NE transporter, and presynaptic αAR feedback, where NPY potently inhibits the release of NE at the prejunctional level. NPY also modulates the activity of bone marrow mesenchymal stem cells (BMSCs), hematopoietic stem cells (HSCs), and chondrocytes, such as regulating BMSCs mobilization. The angiogenesis of endothelial cells (EC) in bone is regulated by NPY and its receptors as well

Due to NPY’s powerful regulatory ability in modulating various cell types, it determines bone formation and resorption rates through multiple pathways, which is crucial for preventing bone structural damage and bone metabolism disorders. NPY is a multifunctional neurotransmitter that acts through five well-studied receptors (Y1R, Y2R, Y4R, Y5R, and Y6R) in mammals, which are categorized as G protein-coupled receptor subtypes.^156,157^ However, different NPY receptors exhibit varying NPY affinities (Y2R > Y1R > Y5R, and Y4R = Y6R).^158^ The NPY-mediated modulation of bone is summarized in Fig. 3. We next review the modulatory functions of reported receptors on skeletal metabolism in detail.

Y1R, involved in modulating bone mass, mitogenic activity, pulpal inflammation, and macrophage migration,^146,159,160^ is predominantly distributed in the CNS’s PVN.^133,161^ Y1R is abundantly expressed in MSCs, osteocytes, monocytes, OBs, OCs, and macrophage lineage cells,^146^ indicating a local NPY regulatory role. Specific Y1R deletion in OBs enhances mineral apposition rate and bone mass,^152^ while germline ablation shows a negative role in bone turnover.^146,162^ Mice treated with a Y1R antagonist^163^ or globally deleted Y1R^164^ exhibited increased differentiation and proliferation of mature OBs and osteoblastic progenitor cells. However, conditional hypothalamic Y1R knockout did not alter bone homeostasis,^165^ suggesting a peripheral inhibitory role. A study found that Y1R antagonist, not NPY itself, elevated BMD upon local hyaluronic acid injection in male rats.^166^ High bone mass and altered extracellular matrix ultrastructure were observed in germline Y1R^−/−^ mice.^167^ Y1R antagonist accelerates microdamage repair and osteogenic differentiation of MSCs via the cAMP/PKA/CREB pathway,^168^ suggesting potential therapeutic application for osteoporosis and osteoporotic fracture prevention. Y1R antagonist also influences gut bacteria and ameliorates ovariectomy-induced osteoporosis in rats,^169^ revealing complex, multiple pathways in bone mass modulation. Depletion or inhibition of Y1R promotes bone metabolism predominantly through peripheral roles, supporting local administration for skeletal disease treatment.

Y2R, on the other hand, is the most highly expressed receptor subtype of NPY in the CNS and accounts for almost two-thirds of NPY binding activity in the CNS. Y2R is primarily located in presynaptic regions and is highly expressed in the hypothalamus, hippocampus, and brainstem.^161^ Additionally, Y2R is found in peripheral tissues such as adipose tissues, muscle, spleen, intestine, and liver.^170^ As a result, the heterogeneous distribution of Y2R dictates multiple biological functions in different organs. Deletion of Y2R in the hypothalamus revealed increased osteoblastic activity and bone mineralization rate, which gradually led to elevated bone turnover, demonstrating Y2R has a catabolic role in cortical and cancellous bone metabolism.^129,171^ Both hypothalamic and germline ablation of Y2R promoted bone anabolism, suggesting Y2R may primarily act centrally to regulate bone formation in adult mice.^153,172^ Moreover, current evidence revealed that conditional knockout of peripheral Y2R showed no significant effect on the regulation of bone mass.^170^ These studies focused on Y2R confirm the importance of the central neuronal pathway in NPY modulation of bone. Consistently, Y2R antagonist treatment in ovariectomized mice increased BMD.^173^ Therefore, NPY regulation through the Y2R receptor should be considered when treating bone diseases.

Global Y1R and Y2R double knockout mice display similar bone features, indicating a shared pathway in bone homeostasis.^165^ However, hypothalamic double-null phenotype of Y2R and Y4R showed higher osteoblastic activity than single Y2R-ablated mice,^174^ suggesting a synergistic Y4R role. Evidence for Y5R and Y6R is limited. Y5R colocalizes with Y1R in the CNS^161^ and influences the self-regenerative capacity of MSCs.^160^ Y6R is expressed in the hypothalamic suprachiasmatic nucleus (SCN),^175^ but its role in the human genome is unclear. NPY accelerates osteoblastic differentiation, proliferation, and prevents MSCs apoptosis via Y1R.^176^ NPY treatment decreases OCs levels by stimulating hematopoietic stem cells (HSCs) mobilization through Y2R and Y5R and facilitates EC migration, proliferation, and capillary formation via Y1R, Y2R, and Y5R.^177^ These findings highlight NPY and its receptors’ relationship with bone metabolism, offering a novel approach for clinical therapy in central sympathetic regulatory pathways.

#### The potential involvement of hypothalamic interoception on bone metabolism via agouti-related peptide

Approximately 95% of AgRP acts as an orexigenic peptide and is exclusively co-expressed with NPY^+^ neurons within the ARC of the hypothalamus.^4^ AgRP drives hunger by suppressing melanocortin 3/4 receptors and inhibiting α-melanocyte-stimulating hormone (α-MSH) signaling in brain tissues, thereby blocking POMC/CART neurons and inducing the inhibition of food intake.^178^ AgRP-deficient mice showed no significant changes in appetite and body weight, while an obese phenotype was observed in AgRP-overexpressing mice.^179^ These findings suggest that AgRP signaling is not essential for maintaining appetite and body weight under basal conditions.^4^ Generally, NPY upregulation in fasting animals is associated with the downregulation of somatostatin and reduction of growth hormone secretion, resulting in decreased serum insulin-like growth factor-1 (IGF-1) and growth hormone levels. Consequently, growth inhibition (body weight, lean mass, and fat) has been observed with NPY upregulation.^180,181^ Simultaneously, appetite reduction has been attributed to the downregulation of AgRP.^180,181^ Therefore, although the major actions of AgRPs seem to involve NPY in appetite regulation, they also exert unique effects on energy homeostasis.

AgRP neurons have also been found to regulate bone mass. It has been determined that uncoupling protein 2 (UCP-2) is expressed in AgRP neurons, and UCP-2 gene deletion impairs AgRP neurons’ function, leading to severe osteoporosis.^182^ In mice, decreased bone mass was observed when the AgRP circuitry was damaged by early postnatal ablation of Sirt1 in AgRP neurons or by cell-autonomous Sirt1 deletion (AgRP-Sirt1^-/-^).^182^ However, the absence of leptin receptors in AgRP neurons did not affect bone homeostasis.^182^ Moreover, inhibition of sympathetic tone in AgRP-Sirt1^-/-^ mice reversed osteopenia.^182^ These findings demonstrate that hypothalamic AgRP neuronal signaling regulation of bone metabolism is independent of leptin-induced sympathetic activity, emphasizing AgRP’s importance in bone homeostasis. A recent study also showed that AgRP signaling negatively modulates bone mass, as evidenced by an AgRP deletion model displaying increased bone mass in trabecular and cortical bone tissues, particularly in male mice.^183^ Furthermore, female AgRP-deficient mice exhibited elevated POMC expression in the ARC.^183^ In contrast, AgRP deletion in male mice has been found to downregulate POMC expression in the ARC without affecting NPY or CART expression, indicating that elevated bone mass in AgRP-deficient mice is independent of NPY signaling.^183^ This is consistent with the finding that bone mass remains unchanged in response to the specific deletion of NPY in AgRP-positive neurons. These findings demonstrate that AgRP potentially modulates bone homeostasis with intrinsic complexity.

#### Hypothalamic interoception interacts with cocaine and amphetamine-regulated transcript (CART) to regulate skeletal metabolism

CART, a neuropeptide precursor, is predominantly found in the hypothalamus and relates to energy balance and appetite.^7^ It is present in various hypothalamic nuclei and co-produced with other appetite regulators like POMC but not with NPY in the ARC.^184^ CART plays a crucial role in bone metabolism, particularly bone resorption, acting downstream of leptin signaling and opposing NPY. CART expression decreases in leptin-deficient mice, accelerating bone resorption.^185^ CART knockout mice show an osteopenic phenotype due to elevated bone resorption.^105^ Increased CART expression is observed in MC4R knockout mice, with CART knockout mice displaying reduced bone mass.^186^ These studies confirm CART’s importance in leptin-mediated modulation of bone homeostasis, highlighting the complex CNS connections regulating the skeletal system.

#### The endocannabinoid system’s role in skeletal interception regulation of bone metabolism

The EC system is a sophisticated endocrine system comprising cannabinoid receptors (CBRs), endocannabinoid ligands, and related biosynthesizing and biodegrading enzymes.^187^ It is also involved in skeletal interoceptive signals in bone metabolism. Endocannabinoids, such as N-arachidonoylethanolamide (anandamide, AEA) and 2-arachidonoylglycerol (2-AG),^188,189^ are the primary endogenous ligands of cannabinoid receptors with high affinity. These endocannabinoids, AEA and 2-AG, can be produced by OBs and OCs in bone^190,191^ and can notably bind with cannabinoid receptor type 1 (CB1R) and cannabinoid receptor type 2 (CB2R).^187^ OBs, OCs, osteocytes, and chondrocytes express both CB1R and CB2R in bone tissues.^192–194^

**Fig. 4:**
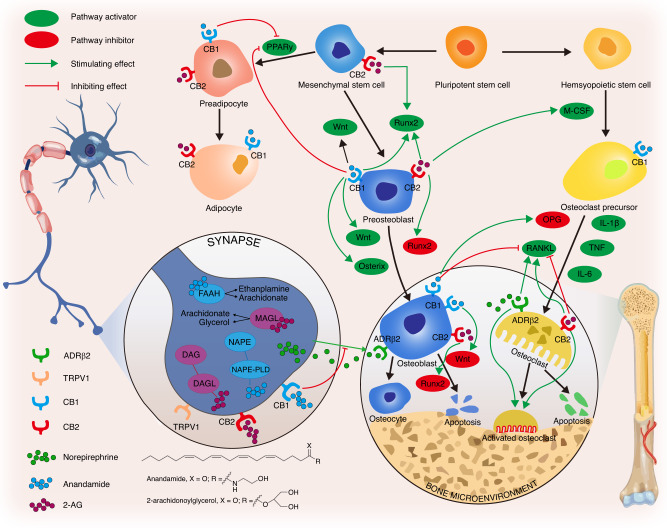
The endocannabinoid system responds to skeletal interoception to maintain bone homeostasis. Anandamide (AEA) and 2-arachidonylglycerol (2-AG) are two endocannabinoids in the bone microenvironment. The CB1 receptor predominantly regulates bone metabolism by acting on sympathetic nerve terminals. Activated CB1 receptors suppress norepinephrine release at sympathetic nerve terminals, inhibiting osteoblast β2-adrenergic receptors, and subsequently increasing osteoblast activity and differentiation. Additionally, inhibited β2-adrenergic receptors reduce osteoclast formation through down-regulating RANKL. CB1 and CB2 receptors are extensively distributed in osteoblasts, osteoclasts, and their precursors, playing crucial roles in maintaining bone homeostasis

The CB1 receptor predominantly regulates bone metabolism by acting on sympathetic nerve terminals. Activated CB1R suppresses the release of NE at sympathetic nerve terminals, thereby inhibiting β2AR on OBs to increase OBs differentiation. Moreover, inhibited β2AR on OCs reduces osteoclast formation by downregulating RANKL.^187,189,194^ CB2R plays a crucial role in maintaining the balance between bone resorption and bone formation. CB2R is highly expressed during the bone remodeling stage of the bone cycle, and its stimulation has a positive effect on OBs proliferation due to increased osteogenic factors, such as RUNX2, bone sialoprotein, alkaline phosphatase, and osteocalcin.^189^ The detailed regulatory roles of the endocannabinoid system in bone metabolism are shown in Fig. 4.

## Traumatic Brain Injury (TBI) disturbs skeletal interoception to induce aberrant bone metabolism

TBI patients exhibit a higher risk of developing osteopenia and osteoporosis, resulting in bone fragility and fractures due to the micro-architectural deterioration of bone tissue. These changes in bone are marked by significant reductions in BMD, attributed to immobility and other metabolism-related factors.^32,195,196^ Similar clinical studies reveal that brain-injured patients displayed an increased risk of hip fracture and reduced BMD due to decreased mechanical loading on bones induced by immobilization following injury, which then activated bone resorption.^197–199^ This has been further verified in animal studies, where TBI reduced BMD in cortical bone and tibial trabecular, irrespective of mobility.^200–202^ TBI potentially affects bone remodeling due to increased sympathetic outflow.^203^ These studies clearly demonstrate that once the CNS is injured, the entire body interacts with the deformation of the skeletal system. TBI-induced skeletal alterations are also attributed to variations in the parathyroid hormone and vitamin D axis,^32,195,204–206^ suggesting a novel pathway through which the CNS can trigger bone metabolism. Long-term loss of bone mass is common following TBI, as it functions through diverse aspects primarily triggered by central pathways. Thus, in this section, we discuss the skeletal interoceptive-related mechanism in the aberrant bone metabolism induced by TBI.

Brain injury has also been shown to accelerate fracture healing. Patients with fractures and TBI experience faster healing, enhanced callus formation, and increased bone volume and BMD.^32,203,207^ Recent clinical investigations concerning TBI’s acceleration of fracture healing are summarized in Table 1. Animal models with TBI and fractures also exhibit improved callus strength and bone volume.^208,209^ The mechanisms behind this accelerated healing remain unclear, but leptin and NPY, reviewed earlier, may play a role. In a rat model, fractures with TBI had higher leptin-positive cells in callus, callus volume, and serum leptin levels than fracture-only rats.^210,211^ Elevated cerebrospinal fluid leptin may result from a disrupted blood-brain barrier and increased serum growth hormone and IGF-1 after TBI, accelerating healing.^212^ NPY levels increased and promoted MSCs osteogenic differentiation in TBI and fracture patients.^213^ TBI-induced bone formation increases were preceded by elevated 2-AG and decreased NE levels, suggesting sympathetic control of bone formation via 2-AG activation of prejunctional CB1.^214^ Contralateral TBI to the fracture site significantly increased bone formation, emphasizing the comprehensive effect of nerves on bone formation regulation.^215^

**Table 1:** Recent clinical research investigating how traumatic brain injury (TBI) accelerates fracture healing

Number of patients	Results and suggested mechanisms	Year and Ref.
Fracture-only: 3 Fracture with TBI: 3 Healthy controls: 3	In the fracture with TBI group, several critical lncRNAs (including ENSG00000278905, ENSG00000240980, ENSG00000255670, and ENSG00000196634) were involved in regulating the cellular activity of basophils, cytotoxic T cells, B cells, and endothelial cells.	2021^216^
TBI: 30 Femoral fracture: 30 Femoral fracture with TBI: 30 Healthy subjects: 30	Faster femoral fracture healing occurred in the femoral fracture with TBI group than in the femoral fracture-only group. BMP-2, FGF-2, IL-1β, and PDGF levels in the femoral fracture with TBI group were significantly elevated over 12 h and after 4 weeks (indicating their potential involvement in accelerated fracture healing among patients with TBI).	2021^217^
Tibial fracture with TBI: 26 Simple tibial fracture: 26	TBI promoted callus formation and heterotopic ossification in patients with fracture but did not alter fracture healing time. In patients with tibial fracture and brain injury, reduced expression of miR‑433 resulted in up-regulated expression of SPP1 in calluses, heterotopic ossification tissues, and plasma.	2021^218^
30 female patients: Completely healed fracture: 10 Fracture with TBI: 10 Isolated fracture: 10	Fractures accompanied by TBI up-regulated the expression of miRNA-92a-3p in systemic circulation, inhibited IBSP levels at the fracture site, activated PI3K/AKT signaling, and accelerated the translation and transcription of osteogenic genes, thus accelerating fracture healing and callus formation.	2021^219^
Fracture: 6 Non-fracture: 6 Fracture with TBI: 6	Patients with fracture and TBI had significantly lower serum miR-16-5p levels at 24 h and 72 h post-injury compared to the fracture-only patients.	2019^220^
Fracture: 6 Non-fracture: 6 Fracture with TBI: 6	Elevated miRNA-26a-5p may be involved in faster fracture healing in patients with TBI by inhibiting PTEN and PI3K/AKT signaling.	2019^221^
Clavicle fracture with TBI: 22 Clavicle fracture alone: 25	Blood vessel formation was increased in patients with fracture and TBI. NGF and VEGF levels were higher in patients with fracture and TBI, as compared to patients with only fracture, contributing to shorter fracture healing time.	2018^222^
Clavicle fracture with TBI: 22 Clavicle fracture alone: 25	Increased percentages of M2 macrophages were correlated with shorter fracture healing time in patients with TBI. In addition, M2 macrophage polarization in bone regeneration was found to potentially promote bone fracture healing.	2018^223^
Long-bone fracture with TBI: 25 Long-bone fracture alone: 33	Shorter fracture healing time in patients with TBI may result from enhanced HIF-1α levels in fracture haematoma and serum.	2017^224^
Limb fracture with TBI: 25 males and 15 females Simple fracture: 27 males and 13 females TBI: 28 males and 12 females Healthy: 24 males and 16 females	Serum NGF and EGF levels were increased in the limb fracture with TBI group and involved in promoting fracture healing.	2013^225^
Femoral shaft fracture with TBI: 20 Femoral shaft fracture alone: 49	TBI was correlated with faster fracture healing and elevated callus formation. TBI severity and intracranial haemorrhage type did not statistically contribute to bone healing.	2012^226^
Mandibular fractures with severe TBI: 24 Mandibular fractures alone: 21	Time to callus formation was positively correlated with TBI and coma duration but not with age, gender, and fracture distribution.	2012^227^

**Fig. 5:**
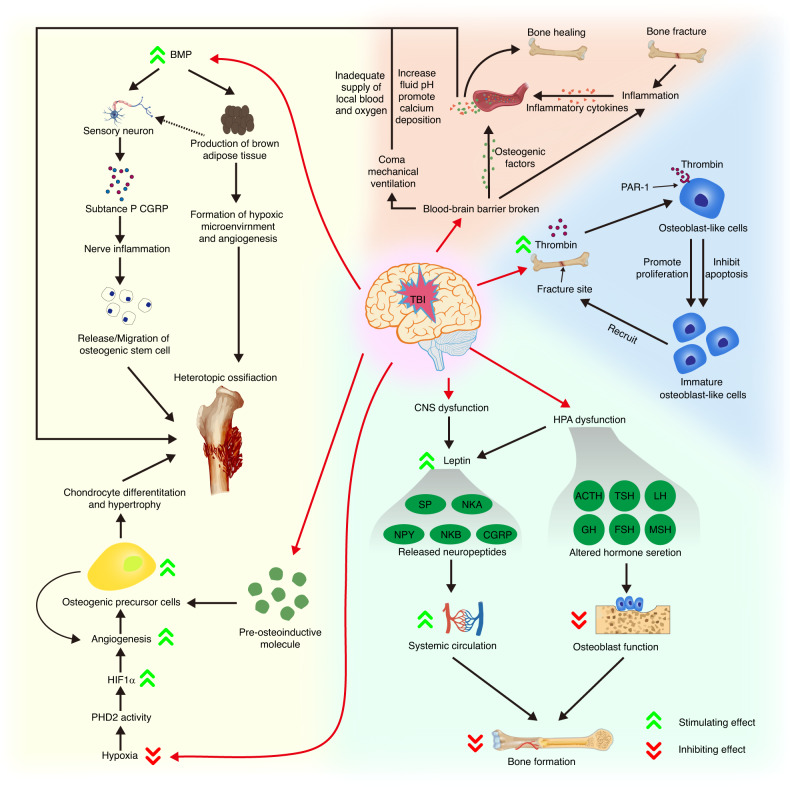
Skeletal interoceptive impact of traumatic brain injury (TBI) on bone formation and heterotopic ossification. TBI disrupts the hypothalamus-pituitary-adrenal axis (HPA) and central nervous system (CNS), leading to elevated leptin levels due to a damaged blood-brain barrier. Subsequently, released neuropeptides, including substance P (SP), neurokinin A (NKA), neuropeptide Y (NPY), neurokinin B (NKB), and calcitonin gene-related peptide (CGRP), inhibit bone formation through systemic circulation via the leptin pathway. HPA dysfunction alters hormone secretion, such as adrenocorticotropin (ACTH), prolactin (PRL), growth hormone (GH), luteinizing hormone (LH), follicle-stimulating hormone (FSH), melanocyte-stimulating hormone (MSH), and thyrotropin (TSH), which inhibit bone formation. Regarding heterotopic ossification (HO), hypoxic conditions and bone morphogenetic protein (BMP) signals are associated with HO development following TBI. TBI-related blood-brain barrier dysfunction triggers the delivery of several osteogenic factors into the bloodstream, promoting bone healing and accelerating HO development. Moreover, TBI-induced hemorrhage leads to thrombin release at the injury site, accelerating osteoblast proliferation and suppressing apoptosis, thus hastening bone healing

Although TBI induces bone loss in a homeostatic state, its significant role in accelerating bone fracture healing may indicate that skeletal interoception signals during TBI have a predominantly beneficial effect on tissue reconstruction. This requires further investigation across multiple pathways to be confirmed, but various studies have already demonstrated a definite link between these two disorders. The detailed regulatory pathways of TBI on bone formation are shown in Fig. 5.

BMP-2, bone morphogenetic protein 2. FGF-2, fibroblast growth factor 2. IL-1β, interleukin-1 beta. PDGF, platelet-derived growth factor. PI3K/AKT, phosphatidylinositol 3-kinase/threonine kinase 1. SPP1, osteopontin. PTEN, phosphatase and tensin homolog. NGF, nerve growth factor. VEGF, vascular endothelial growth factor. HIF-1α, hypoxia-inducible factor-1alpha. EGF, epidermal growth factor. IBSP, integrin binding sialoprotein

## Summary and future study

Skeletal interoception mechanisms in bone homeostasis regulation are complex, involving central modulation primarily targeting OBs, OCs, and osteocytes. The hypothalamus acts as the regulatory center through the autonomic nervous system, neuropeptide release, and neuroendocrine mechanisms, controlling MSCs differentiation, OCs activation, and bone cell functionality. Sensory nerves innervate skeletal tissues, enabling the CNS to receive bone-derived signals and maintain homeostasis through various responses. OBs-derived PGE2/EP4 signals play a key role in skeletal interoceptive pathways, regulating MSCs differentiation and bone mass accrual via the sensory nerves-hypothalamus-sympathetic tone axis. Further investigation is needed to understand the regulation of sympathetic and sensory nerves on bone development, mass accrual, and remodeling. The complex regulatory mechanisms and CNS-peripheral nervous system interactions on bone physiology warrant in-depth exploration. The effects of skeletal interoception on brain-bone crosstalk are not well understood, but communication between these organs is essential for bone homeostasis. More research is needed to deepen the understanding of bone development and fracture healing mechanisms and facilitate clinical applications.
